# Selections and Abstracts

**Published:** 1914-11

**Authors:** 


### SELECTIONS AND ABSTRACTS

### UNITED STATES CIVIL-SERVICE EXAMINATION. EPIDEMIOLOGIST, MALE ($4,000).

December 15, 1914.

The United States Civil Service Commission announces an open competitive examination for epidemiologist, for men only. From the register of eligibles resulting from this examination certification will be made to fill a vacancy in this position in the Public Health Service for service in the field, at a salary of $4,000 a year, and vacancies as they may occur in positions requiring similar qualifications, unless it is found to be in the interest of the service to fill any vacancy by reinstatement, transfer or promotion.

The duties of this position will be to make laboratory and field investigations of the diseases of man in relation to prevalence, causation and methods of control, and to conduct field studies of public health matters. It is desired to secure persons thoroughly qualified to do laboratory and field research work in epidemiology, and to organize and conduct such work in the field.

Competitors will not be assembled for examination, but will be rated on the following subjects, which will have the relative weights indicated:

| Subjects. | Weights. |
|---|---|
| 1. Education | 25 |
| 2. Experience and fitness | 50 |
| 3. Publications | 25 |
| Total | 100 |

Graduation with an A. B. or B. S. degree from a college or university of recognized standing, and graduation with an M. D. degree from a medical school of recognized standing, and at least five years' experience in epidemiological research, including field studies and laboratory technique, and at least five years' public health service under Federal, State or municipal authorities, are prerequisites for consideration for this position. Experience in epidemiological research and experience in public health service may be concurrent.

Statements as to education and experience are accepted subject to verification.

Applicants must have reached their twenty-fifth but not their fortieth birthday on the date of the examination.

This examination is open to all men who are citizens of the United States and who meet the requirements.

Persons who meet the requirements and desire this examination should at once apply for Forms 304 and 2095, stating the title of the examination for which the forms are desired, to the United States Civil Service Commission, Washington, D. C., the Secretary of the United States Civil Service Board, Post Office, Boston, Mass., Philadelphia, Pa., Atlanta, Ga. Cincinnati, Ohio, Chicago, Ill., St. Paul, Minn., Seattle, Wash. San Francisco, Cal.; Customhouse, New York, N. Y., New Orleans, La., Honolulu, Hawaii; Old Customhouse, St. Louis, Mo.; or to the Chairman of the Porto Rican Civil Service Commission, San Juan, P. R. No application will be accepted unless properly executed, excluding the medical certificate, and filed with the Commission at Washington prior to the hour of closing business on December 15, 1914.

Issued November 11, 1914.

---

### THE TREATMENT OF HEART INVOLVEMENT IN SYPHILIS

### BASED ON A STUDY OF THREE HUNDRED CASES*

Harlow Brooks, M. D., and John Carroll, M. D.,

New York.

We have already written concerning the great frequency of heart involvement in syphilis.[1] Here and elsewhere,[2] we have discussed the character of the changes in the organ, pointing out that the most frequent and important lesion in all stages of the disease is one of the heart-muscle which is dependent on a periarteritis of the coronary vessels. This lesion was apparently first described by Isaac Adler,[3] and its frequency of occurrence, since noted by many, has been recently verified by Warthin,[4] who has demonstrated with considerable constancy the presence of spirochetes in these characteristic foci.

In another paper,[6] presented as a continuation of our study of this subject we have discussed the symptoms and diagnosis of the complication.

---

*From the Second Medical Service of the City Hospital.

*Read before the Section on Practice of Medicine at the Sixty-Fifth Annual Session of the American Medical Association, Atlantic City, N. J., June, 1914.

1. Brooks, Harlow: A Study of the Heart in Syphilis, Med. Rec., New York, 1912, lxxxi, 351.

2. Brooks, Harlow: Clinical Observations Concerning the Heart in Syphilis, Inter-State Med. Jour., 1913, xx, No. 6; The Heart in Syphilis, Am. Med. Jour. Med. Sc., 1913, cxlvi, 513.

3. Adler, Isaac: Observations on Cardiac Syphilis, Tr. Assn. Am. Phys., May 3, 1898.

4. Warthin, A. S.: Tr. Assn. Am. Phys., May 13, 1914.

5. Brooks, Harlow, and Carroll, John: The Symptoms and Diagnosis of Involvement of the Heart in Syphilis, Based on a Study of Two Hundred Cases, New York State Jour. Med., 1913, xiii, 328.

Some of these cases have now remained sufficiently long under observation to permit us to discuss with some authority the question of treatment and its results, covering cases in the secondary and tertiary stages of the disease. It is our purpose to present in this paper certain aspects of this important side of the subject in so far as our rather brief though active experience now goes.

We have found it very difficult to discuss treatment in an analytical way inasmuch as so many cases have come under our observation on our hospital services, where definite data could not be elicited as to the precise methods of specific treatment previously employed, nor of its extent.

Obviously in a large percentage of the cases the time at which the heart-lesion developed could not be ascertained; in fact, this point could be surely fixed only in some of the secondary cases. The character and extent of previous treatment for circulatory discrepancies can also not be definitely determined.

We have included in our list for analysis a number of cases which have been treated in various hospitals or by private physicians with entire disregard of the luetic origin of the cardiac fault. Such instances have been included because we have found that comparison of these with those treated on the assumption of a definitely syphilitic change affords a basis for conclusions of value. Only four instances are found, however, in this series in which improvement without specific treatment took place.

Since a considerable number of cases have only been conclusively recognized by lesions discovered post mortem, it must be understood that it is highly probable that the percentage of fatal results in this group of three hundred cases is overestimated, and it is therefore not mentioned, as having no accurate bearing on the treatment of this complication. The apparent recovery or signal benefit of equally desperate cases, when treated on the basis of the luetic origin of the heart defect, certainly indicates the necessity of such a therapeutic basis. We are keenly aware that so crude an analysis as is possible on material of this character does not establish a certain scientific fact, but we feel that our study, covering, as it does, over three hundred cases, has led us to conclusions of very real applicable value from the clinical point of view.

It will be noted throughout our methods of treatment vary greatly, and that no one routine type of drug or procedure has been adhered to. This may be in part due to the fact that our therapeutic studies have been, and still are, in an experimental stage, but chiefly because we have been driven to the conclusion that in the treatment of patients suffering from this complication each instance must be definitely individualized, and that no one routine method will be found to apply with equal benefit in all cases. Unscientific as this conclusion may appear to some, to those long actively concerned in the treatment of disease in routine patients, it needs no defense, and the absolute necessity of such a basis for the best therapeutic results will be apparent.

We again wish to emphasize the fact that the essential lesion, in so far as the heart in syphilis is concerned, is a periarterial granulomatous or inflammatory change affecting the heart-muscle. From the standpoint of treatment, we believe that this assumption is most important, for our studies have shown the final or immediately lethal lesion in most cases to be a giving way of the muscle. Furthermore, we are convinced that in most cases where improvement has followed treatment it has been accomplished by a clearing up of these rather than of other lesions, though we recognize with Sachs, Collins and many others after them that a true endocarditis, even of considerable standing, may be absolutely removed by specific treatment.

We also wish to state that in our opinion some of the most brilliant results attributed to specific treatment in cases of syphilitic aortitis have in reality been achieved from improvement of the universal heart-muscle lesions, for many of the symptoms commonly attributed to this lesion are really due to muscle disease. This assumption is apparently supported by the recent pessimistic but undoubtedly accurate studies of Warthin.

Case 1 (231).—M. F., woman, aged 50. Negative history as to lues. Five years ago, epigastric and precordial pain with vomiting, dizziness, headache and weakness. Shortly after, dyspnea on least exertion. One year later edema, precordial pain reflected down left arm into hand. All symptoms recently greatly increased. Physical examination showed poor muscle tone with systolic at mitral and at aortic rings. Broad area of retrosternal dulness, tenderness over manubrium. Roentgenoscopy shows dilated arch. Under circulatory treatment in hospital for three months, constantly losing ground, all symptoms progressing. Wassermann reaction negative. One grain mercuric salicylate given intramuscularly with idea of activating Wassermann. Marked improvement in pain within twenty-four hours. Circulatory drugs discontinued and mercuric salicylate continued in vigorous dosage. Patient now up and about ward, apparently free from all symptoms except on exertion. Wassermann reaction positive. Roentgenoscopy shows no apparent change in aortic shadow.

TREATMENT IN SECONDARY CASES

The early cases treated have mostly occurred either in the private practice of one of us (Carroll), or in the service of our colleague, Dr. J. A. Fordyce, who has greatly encouraged our studies of this subject. Twenty-four such instances have been observed. Two fatalities occurred among them (previously reported) but in both the nature of the disease and its complication had not been recognized in sufficient time to admit of proper treatment. Confirmatory necropsies were secured in both. Apparently complete recovery without any subsequent heart defect took place in all the other cases, save one to be mentioned later. We wish in this regard to point out the care which should be exercised by those in charge of early syphilis that cardiac strain be avoided until treatment has been established, and that the same necessity for consideration of the circulatory apparatus be recognized as in the other infections, diphtheria, rheumatism, septicemia and the like.

Case 2 (199).—Man, aged 34. Service of Dr. J. A. Fordyce. In secondary stage of the disease. Complains of shortness of breath, sudden attacks of precordial pain. Chills, fever, leukocytosis and the physical signs of pericarditis. Complete disappearance of signs and symptoms within three days after two doses of neosalvarsan (intravenously). The cardiac signs appeared within four weeks after initial lesion, which in this case was on nose. Apparent cure followed. Subsequent combined treatment.

Although practically all the early cases under our observation have been treated by the more modern methods, that is, with salvarsan, we are convinced that vigorous treatment with mercury in this stage would probably also result in almost as brilliant results. We wish to assert that, aside from general prophylactic measures, the treatment of cardiac invasion in early syphilis should be directed only to the specific infection and that circulatory treatment per se is at this time unnecessary and probably unwise.

Although we are convinced that in by far the greater majority of cases of early lues cure of a cardiac complication may be confidently expected, certain exceptions exist. This has been well illustrated by a case referred to one of us by Dr. Alphonse Wren.

Case 3.—Patient, woman of 25. Came under observation in the aggressive secondary stage of the disease. She had been treated, apparently mostly with mercury and the iodids in various dispensaries and by private physicians. In spite of treatment exhibits mucous patches, marked skin rash, periosteal tenderness, fever, falling hair and other evidences of active secondary lues. While still under vigorous treatment with mercuric chlorid internal, mercurial inunctions, and after three doses of salvarsan carefully administered in an excellent clinic, developed cardiac muscle insufficiency, dilatation, active aortitis (without aneurysm) endocarditis and edema and cyanosis with severe dyspnea. Salvarsan intravenous again repeated in maximum dose. Mercuric salicylate injected intramuscularly until marked salivation and other evidence of mercurial poisoning appeared. Digitalis, strophanthus, camphor and the usual cardiac regimen apparently produced no effect whatever. The case was progressing rapidly and absolutely unchecked by either specific or circulatory measures when she passed out of our observation. Though her subsequent history cannot be ascertained, it is impossible to assume other than that early death followed.

In so far as could be judged by study and observation of this case, treatment of all kinds was entirely without avail in so far as checking the course of the infection or relieving the heart complication was concerned. This has been the only case in our observation in which treatment in the secondary stage has been apparently useless. Inquiry among syphilographers of wide experience has brought to light other and probably similar cases. They would, however, appear to be quite unusual.

### TREATMENT IN LATE CASES.

Most of the cases comprised in this series are those occurring, or at least presenting themselves, on an internal service for treatment in the tertiary or late stages of the disease; 277 instances of this type are included.

A few patients appeared for treatment for the first time because of their cardiac condition; but in most instances, and in practically all cases which came to our hospital service, they had been for a greater or lesser time under treatment either by private physicians or in institutions for their circulatory discrepancies. While in some of these cases a certain amount of benefit had been derived from such measures, mostly applied entirely without recognition of the cause of the circulatory defect, the very fact that they appeared for treatment shows that these measures in themselves had been found inadequate, probably even at the very outset of the complication. As illustrative of this point we wish to present briefly the case of a fire captain 52 years of age.

Case 4.—He came to one of us suffering severe precordial pain and so marked a grade of dyspnea that he was hardly able to get about. He had been ordered before the retiring board because of physical disability. On examination he showed loud, double aortic murmurs, and irregular and considerably dilated heart, together with the symptoms of a probable aortitis. The man gave a history of a probable rheumatic fever but denied that of syphilis. He was placed on digitalis, but without good effect. Because of the failure to respond, and also because of the evidence of an old tibial periastitis, although a Wassermann had been refused, he was taken off the cardiac medication though, of course, circulatory precautions were still insisted on. He was tentatively placed on protiodid of mercury by mouth. Within a few days such marked improvement had taken place that when the patient was confronted with the facts of the case he admitted a syphilis twenty years ago, which had been at that time briefly treated. A continuation of the same line of treatment with the protiodid of mercury, assisted by potassium iodid, has resulted in complete disappearance of the dyspnea and other evidences of cardiac incompetence. The double aortic murmurs have practically disappeared and he has been doing full duty for some twelve months and is today in comparatively complete health, although still under treatment with the mercury.

These two facts stand out in this illustrative case: circulatory medication was entirely without avail, complete circulatory relief was afforded by the simple administration of mercury.

Notwithstanding frequent brilliant results of this kind, in tertiary instances we have found it by all odds most advisable to also place the patient under the usual hygienic and therapeutic regimen indicated by the circulatory condition. Those cases which, independent of the cause, should be put at rest or in bed, should be, of course, so managed. Furthermore, when digitalis, strophanthus, camphor, strychnin or epinephrin seem to be indicated, they should be given. We have found, however, that in most instances these measures do not give the expected amount of improvement, except for that directly due to rest, in whichever form it may be decided, unless and until the patient has been under the effects of specific medication of one or another variety. Not at all infrequently, as in the instance cited above, such a measure is at once followed by so marked benefit that the drug measures designed for circulatory compensation may be advantageously discontinued. In long-standing cases, however, particularly those in which it may be fairly assumed that fibrosis has taken place in the heart-muscle, the use of digitalis and its adjuvants or substitutes is absolutely necesary. A fact most vividly shown, however, is that these drugs used alone fail in most instances to give good results unless combined with the specific methods.

Although immediate improvement may follow the introduction of specific medication, it has been amply demonstrated in this, as in all other types of luetic disease, that for permanent results or even for steady progress, the specific medication must be persistently carried out. With a discontinuation, such as has taken place frequently when patients insist that they are sufficiently well to leave the hospital, and probably rarely then continue to carry on the treatment in anything like an adequate manner, relapses are apparently almost certain to occur. We have noted that when the treatment has been thus interrupted when it is resumed rarely indeed does a prompt response to it occur comparable to that first manifested. In other words, a tolerance of the lesion against the drug seems to have become established in some cases.

Case 5 (67).—Man, aged 51. Chancre at 30 years. Local treatment only. Present illness began in 1908. Chief complaint, dull, boring pain in left chest associated with dyspnea. For two years a constant increase in distress. Has been under almost constant circulatory treatment without relief. Wassermann positive. Given 1 grain mercuric salicylate hypodermically every 5 days. Pain, dyspnea and edema began promptly to subside. Treatment continued for nine months (sixty-six injections); three doses of salvarsan also given intravenously together with various symptomatic measures. Patient able to be discharged and to resume work as cook after three years of total invalidism. Two months later, treatment having been discontinued, symptoms reappeared, to disappear promptly under resumption of the mercury. The subsequent history has been a return of symptoms whenever specific treatment has been discontinued. Circulatory measures are now necessary to reach the results previously easily obtained by purely specific drugs.

Where the patient can be closely followed and treatment persistently enforced, progress seems to be steady to the point of apparent cure in no inconsiderable number of cases, as indicated by the fact that of the series of 300 cases so treated 116 have shown apparently permanent benefit if not actual cure. The most impressive fact of all is that a large number of cases which appear apparently hopeless go on to clinical cure.

### TREATMENT WITH MERCURY.

As to the precise method of specific treatment which meets with best success in these complications of syphilis, no conclusion appears as yet possible to us except that, at least in most instances, it is a matter of individualization. In not a few cases best results seem to follow an initial vigorous administration of mercuric salicylate given intramuscularly, and in general this would appear to us to be on the whole the most desirable method at the outset. The drug should be pushed to its full point of toleration, but may be subsequently replaced by mercury in the form of the protoiodid or bichlorid introduced by mouth. In some cases, as in an instance already cited, the protiodid appears to be very desirable. It seems to us that such instances are those which do not manifest the most aggressive and active symptoms and, though we are well aware that this form of mercury does not enjoy a good repute with syphilographers in general, it has given us excellent results in many of these heart cases.

Mercuric chlorid has also given us good effects in some instances, either given by mouth or by hypodermic administration.

Case 6.—J. D. Seen seven years ago with Dr. C. J. Imperatori. Patient a powerful, rather obese man, aged 26. Patient unconscious, complete anuria, heart enormously enlarged.

History of long standing, apparently progressive double aortic lesion with very unsatisfactory response to digitalis, etc. Apparently in extremis. He had denied lues, but because of social evidence to the contrary he was given vigorous dosage with mercuric chlorid by mouth and inunctions over precordium of blue ointment. Within a few hours return of renal activity and consciousness, with subsequent cessation of precordial pain and reappearance of regular, full pulse with marked double aortic murmurs and strong, vigorous muscle contractions. Mercury continued with occasional digitalis, which now acted in a proper manner. Given potassium iodid with continued benefit. He largely disappeared from observation up to last year, when he returned with a history and condition of relapse which had developed apparently from discontinuance of treatment and from moderate dissipation. He had meanwhile carried on his work and lived an ordinary life. Relief again followed resumption of treatment but he suddenly died some four months later, apparently from acute dilatation after intercourse.

Inunctions of mercury ointment given in the usual courses also seem to act very well in many cases. In one notable instance we found little result and a very poor toleration of mercury until the old-fashioned, and to our mind usually uncertain, treatment with vapors of calomel was instituted. In practically all instances we have received best results when the form of treatment or of the drug was from time to time altered but, on the other hand, actual interruption of treatment inside of two years for no more than a few months has been followed frequently by relapse and a subsequent unsatisfactory response to this drug. We are anxious none the less not to give the impression that we believe that every case of late syphilis with heart complications will respond to mercurial treatment, and to emphasize this point we cite the following case:

Case 7.—V. H., man, aged 51. An irregular, though shrewd, practitioner in venereal diseases. Lues contracted twenty years ago. Was very aggressively treated at that time with inunctions and by "mixed treatment." Apparent cure. Subsequent life often dissipated. Late in 1913 began to be troubled by anginal attacks, dyspnea, cyanosis and spontaneous attacks of dizziness. Wassermann reaction found to be positive. Diagnosis of luetic myocarditis, adhesive pericarditis, aortitis. Varied mercurial treatment promptly and vigorously resumed, associated with occasional use of iodids. After this some, though unsatisfactory, response to digitalis, strophanthus, and epinephrin, all of which had been previously found inert. Wassermann became negative. Patient absolutely refused to permit the use of salvarsan, saying that he preferred to die rather than to submit to it. Soon died, with the ultimate typical picture of a myocardial degeneration.

TREATMENT WITH SALVARSAN.

Salvarson has been employed in the treatment of seventy-five of this series. In the acute or secondary stage, used independently of mercury, it has in nearly every instance been followed by immediate improvement of the compromised circulatory mechanism, even in patients in whom the circulatory defect appeared to be serious. In such cases it may be that complete cure might follow the use of this drug alone. In all, however, a follow-up course of mercury has been prescribed; some instances observed by us, but not included in this analysis in which salvarsan alone has been employed have not yet remained a sufficient time under observation to allow us to judge as to the permanence of such apparent cures. These brilliant results are much less frequently apparent in later cases, however, and such is our confidence in and reliance on mercury that in no cases have we ourselves continued the use of salvarsan alone.

Our experience has indicated to us that, while no doubt salvarsan gives a most prompt response, its permanency in ter-
does not appear to be established unless the treatment is fol-
tiary complications, referring entirely now to those of the heart,
lowed up by the protracted use of mercury. Where very prompt response to treatment seems to be necessary salvarsan is given, but in cardiac even more than in other complications of this infection a very serious degree of reaction may follow the giving of salvarsan and more than seldom we have been greatly alarmed by circulatory collapse which has followed the administration of salvarsan in those cases especially in which symptoms and signs indicated particularly aggressive invasion of the heart-muscle.

We have been impressed with the observation that those cases which showed disturbances of rhythm as a result of the myocardial invasion are most likely to exhibit serious reaction after the use of salvarsan. We have therefore esteemed it to be good practice to begin the use of arsenic in these cases of heart complication by small doses, allowing the subsequent ones to be larger if no serious grade of reaction is excited. Of all the cases treated by us there were none in which any grave circulatory disturbance threatened as an apparent result of a maximum initial dose of mercury.

As a natural result of these observations we have in questionable cases, and when time did not seem to be of paramount importance, usually first placed the patient on mercury, giving the salvarsan later. We are of the impression that best results are often reached when the salvarsan injections are widely spaced in the course of routine mercury administration. The later injections of salvarsan are much less likely to give serious disturbance than the earlier, especially the first one. Of course it goes without saying that salvarsan is administered only as intravenous injections. It is a very difficult matter to judge, nor do we feel that our cases have been sufficiently numerous to permit of definite conclusions, but we are of the opinion that the old salvarsan is more efficacious in the relief of heart complications in syphilis than neosalvarsan. We have not been able to see that the newer form of the drug produces any less serious collapse than the old in those cases in which this incident arises.

### THE IODIDS.

We have extensively employed the iodids, mostly potassium iodid, in the treatment of these cases of heart invasion. In no instance, however, have we been able to convince ourselves that it acts in any way as a specific. Nor does it, according to our observation, check the progress in an aggressive case. For illustration, we have not found in cases of circulatory deficiency when drugs of the digitalis group failed to act, that results were obtained by combining the administration of the iodid. This, as we have already stated, is to be fully expected, especially in previously untreated cases both with mercury and salvarsan. We have not, however, tried out the iodids alone in cases of secondary syphilis in which heart involvement was present; hence, perhaps, we are not justified in definite conclusions, but we do not believe that this drug is to be considered as a specific in the treatment of active lues.

In tertiary cases, however, there is no doubt whatever in our mind but that the iodids materially assist in the clearing up of the infiltration and exudates, and their effect on gummatous tissue is too well established to admit of question. The point which we wish to make is that while it is most useful in the absorptive stages of treatment, it does not act as a true specific. Iodin should be used after the case has been thoroughly mercurialized, or after salvarsan has been liberally given, and not before or during the early exhibition of these drugs.

### THE EFFECT OF TREATMENT.

That great clinical improvement takes place in most cases and a certain degree of benefit, though perhaps temporary, in practically all, is the most gratifying fact shown by this study. We are entirely convinced that as definite and brilliant results follow proper treatment in cardiac syphilis as in gumma of the brain, in syphilis of the bones or even in the local lesions of the skin and mucous membranes in the same stages of the disease. As to the question of permanence, we are not so certain, comparatively. This is quite as must be expected from the crucial nature of the organ and the very limited degree of restitution and compensatory hyperplasia of a functional type which may take place in lesions of the heart-muscle.

In regard to the manner in which the curative drugs act, we have but little absolute anatomic evidence, for in but two patients who died after what we have considered adequate treatment have necropsies been secured. Sections of the heart-tissue in these two cases (one reported[6]) showed a total absence of the characteristic perivascular cell hyperplasia and infiltration, or of active arteritis in the walls of the coronary vessels. In both instances, however, insulae of fibrosis were found isolated and perivascular in position which still showed evidence of fibrous hyperplasia, just as in simple fibrosis, but in neither were any lesions present which could be justly spoken of as luetic. In neither instance were we able to demonstrate spirochetæ. In view of the report of Warthin already mentioned, however, we are not prepared to say that they might not have been present and our failure to demonstrate them may have been due to technical deficiencies; we can only say that anatomically, as clinically, all evidences of active syphilitic disease was wanting, although the scars or results of course remained.

In incompletely treated cases which came to necropsy we have without exception been able to demonstrate active lesions of a quite characteristic and unmistakable type.

In common with most who have recently studied large groups of any type of syphilitic disease we have not infrequently seen cases in which apparent clinical cure had taken place and yet in which a positive Wassermann reaction could be secured after a sufficient period of interruption of treatment had elapsed. We have looked on these instances as examples of probably cured syphilis in which a persistent Wassermann still remained. This opinion has already been expressed by us in a former paper.

We have had abundant evidence of the fact that the fibrosis which follows on or occurs with the hyperplastic and degenerative alterations of the heart in syphilis are not removed by treatment; further, we are of the opinion, difficult to entirely

6. Brooks, Harlow: Am. Jour. Med. Sc., 1913, cxlvi, 513.

7. Brooks, Harlow: Clinical Observations Concerning the Heart in Syphilis, Interstate Med. Jour., 1913, xx, No. 6.

prove, that this fibrosis may continue to progress even after the primary etiologic factor may have been entirely eliminated. Such is the general trend in fibrosis of non-specific nature, and we are inclined to account on this basis for those cases which, while greatly improved or arrested by the treatment, still, in so far as cardiac fibrosis is concerned, continue to progress just as in those cases in which the lesion may have been excited by a rheumatism, a nephritis or the like. There is then a large group of cases in which definite limitation of actual cure is present.

### THE LIMITATIONS OF TREATMENT.

We are of the opinion that the limit of benefit for specific treatment in these conditions is commonly reached in tertiary or late cases, when the activity of the actual syphilitic process has been checked or cured and when thereafter by the use of the iodids a maximum degree of absorption and tissue restitution has followed, and when on top of this a maximum effect has been produced by the various drugs and methods designed for circulatory improvement.

Some such cases probably demand the occasional administration of mercury or arsenic. We have been unable in several such instances, however, to go beyond a certain point in beneficial treatment, and when in these the circulatory drugs begin to fail us, the downward progress is, in our observation, rapid, nor is it to be checked by any form of medication, specific or otherwise. The patients then assume precisely the same position as late cases of myocardial degeneration or fibrous substitution, such as may develop in the course of many other diseases. At post mortem these instances commonly show extensive patches of myofibrosis, or advanced disease of the coronary arteries with consequent muscle ischemia.

From our somewhat limited experience with cases of acute secondary syphilitic involvement of the heart, we are of the opinion that no limitation of result exists short of true cure except in those rare cases, similar to the one cited, in which specific drugs are entirely powerless. We are of the idea, it is true from a clinical basis only, that complete and absolute cure is to be expected in this stage of the complication.

### WHEN SHOULD SPECIFIC TREATMENT BE ENTIRELY DISCONTINUED?

The answer to this question is most difficult. Obviously in some instances it must be continued after a negative Wassermann has been obtained; for we frequently find patients in whom activity is evident and yet who give a negative Wassermann. We have also noted above that we occasionally find patients still presenting a positive reaction who otherwise appear to be cured.

From our experience in the treatment of syphilis in other departments of medicine, as well as in circulatory involvement, it is obvious that medication of a specific character must be continued for a considerable time, at least one year, after all signs of activity have subsided. We believe, however, that better results are obtained by periodic rather than continuous medication in the final stages and also by variation of the type of the drug or in its form of administration.

We think that treatment should be continued just so long or longer as it is apparent that benefit is being reached either by the specific drug or by the adjuvant employment of a circulatory stimulant. We further believe from our clinical observation, and from our own as well as the recent anatomic studies of the heart-muscle in syphilis by Warthin, that antispecifics should be from time to time given throughout lifetime in certain cases. It is obvious that just so long as any evidence whatever of luetic activity in any part of the body is manifest, treatment should be vigorously pushed, though periods of interruption may be elected so that disease resistance toward the drug falls off.

### THE SUBSEQUENT MANAGEMENT OF THE CASE.

We have just pointed out that, except in acute secondary involvement, we feel that antiluetic drugs may be occasionally beneficially exhibited throughout life even though they may not perhaps be directly indicated by the immediate appearance of the case itself. Aside from this, the subsequent management of the case should be essentially along circulatory lines and it should be carried out with the fact well borne in mind that practically certainly there are foci of fibrosis in the heart-walls, changes in the endocardium and in the walls of the coronary vessels which are in all probability irremediable and permanent.

This recognition appears particularly important in the determination of the proper hygienic management of the case, especially in regard to selection of occupation or sports and in the limitation of such habits as that of smoking. In late cases it can certainly never be assumed that we are dealing with a perfectly normal heart no matter how complete the cure may seem to be. While we make this statement, based on our considerable necropsy experience with these cases, from clinical observation alone one might well assume that complete cure even in some late and advanced cases had taken place. We do not feel, however, that any of these apparent cures have yet remained a sufficient number of years under observation to overthrow the lesions of pathologic anatomy.

### THE PROGNOSIS.

In acute cases, and in the secondary stage particularly, we believe that except in those instances in which resistance to the specific drugs is shown, a good prognosis as to cure may be given.

In cases in which incomplete or brief specific treatment only has been given, we believe that almost without exception great improvement may confidently be promised even in many instances in which circulatory measures alone show little benefit. When, after the patient has been thoroughly placed under the effects of the specific medication, the circulatory drugs still fail to act, the prognosis as to cure or further improvement is definitely bad.

Nevertheless, a bad prognosis as to relief or a considerable period of reasonable comfort and usefulness is never justified until the combined treatment has been thoroughly tried out.

No more gratifying results are reached in chronic cases of any disease than are often shown in cases of this type, apparently at first thought altogether hopeless.

The prognosis in cases which have relapsed after marked or reasonable improvement is not good. Disturbances of rhythm particularly which have failed to show early and definite improvement under treatment rarely justify a good prognosis.

CONCLUSIONS.

1. Cases of heart involvement in early syphilis may be fully cured irrespective of the character of the lesion by vigorous specific treatment alone and independent of circulatory measures.

2. Even well-established and late cases usually respond to treatment with cure, or marked though perhaps temporary benefit.

3. In most tertiary instances purely circulatory measures produce but slight benefit unless preceded by or combined with specific medication.

4. Interrupted or inefficient treatment establishes an immunity or resistance on the part of the lesions against the specific drugs employed. Hence the importance of vigorous and carefully systematized treatment.

5. The most satisfactory treatment is one which combines the use of salvarsan with mercury and the iodids. Combined treatment may be unnecessary in early cases but it is essential in late ones.

6. Salvarsan, preferably old salvarsan, produces in most instances the quicker results. It is capable, however, of inducing serious symptoms and in untried cases of heart involvement it should be administered in small doses until its action has been ascertained, particularly in those lesions characterized by disturbances of rhythm.

7. Mercury alone may produce apparent cure. Best effects are secured with this drug when its form is from time to time changed. Its use appears to be indispensable in all well-established cases.

8. The iodids are valuable adjuvants in the treatment of these cases, especially in their late stages, but they are apparently without specific action.

9. Permanent injury to the heart must be assumed to have taken place in late cases, even though prompt response to treatment and apparent cure occurs.

10. Successful treatment rests primarily on the recognition of the cause of the disease and on its specific treatment. —Journal A. M. A.

44 West Ninth Street—50 West Ninth Street.

---

### THE FOUR COMMON TYPES OF HEART-DISEASE

### AN ANALYSIS OF SIX HUNDRED CASES.*

RICHARD C. CABOT, M. D., BOSTON.

To classify cases of disease according to their pathogenic agent or process, and not solely by naming the region affected or the function disturbed, is the ideal of scientific progress in medicine.

But until the last decade we have made little advance in this direction as regards the diseases which gravely disturb heart function. Thus we still find in standard text-books a section devoted to "mitral regurgitation," its diagnosis, prognosis and treatment, although mitral regurgitation is almost as vague a phase as "spinal paralysis" or "brain fever." Just as a "spinal paralysis" may be due to trauma, to the tubercle bacillus, to the Spirochaeta pallida, to the organism of poliomyelitis or to cancer, so "mitral regurgitation" is only a symptom caused by the action of streptococci, by the degenerative lesions of arteriosclerosis, by the muscle-tiring resistance of nephritic hypertension and probably by many other causes. It probably

*Read before the Section on Practice of Medicine at the Sixty-Fifth Annual Session of the American Medical Association, Atlantic City, N. J., June, 1914.

contributes its mite to the downfall of cases of pernicious anemia, typhoid fever and puerperal sepsis. Hence there can be no rational prognosis or treatment of mitral regurgitation but only of the various underlying diseases in which this leak is a symptom.

A similar criticism applies to all diagnoses of "myocarditis." The micro-organism of rheumatism and of syphilis, the ravages of arterial disease and perhaps many other causes may produce the lesions of chronic fibrous myocarditis, with or without recognizable symptoms. A diagnosis of myocarditis is like a diagnosis of "ulcer;" it calls for an etiologic qualification, such as syphilitic or tuberculosis.

The matter has many practical aspects. A sane prognosis and treatment of "aortic regurgitation," for example, depends on knowing or guessing what disease has produced it. Even physical diagnosis may have to await an intelligent interpretation of its results until we make up our minds what micro-organism is at work, in the heart, as well as elsewhere in the body. When we hear a low, rumbling presystolic murmur at the heart's apex and an early high-pitched diastolic along the left sternal margin, our conclusions about the mitral valve depend on our answer to the question, "Is syphilis or 'rheumatism' at work here?" If the former, then the mitral valve is probably sound; if the latter, probably diseased.

Four relatively new tests have now made it possible—as I think—to reclassify cardiac lesions more satisfactorily: the Wassermann reaction, the "red test"[1] of renal function, the measurement of blood-pressure and the roentgenography of the chest. When auscultation, percussion and the older methods of urinary examination leave us in the lurch, the four diagnostic methods just mentioned can often guide us aright.

With the help of these methods I have attempted a classification of 600 recent hospital cases in which a failing heart was the most notable feature. The results are shown in the accompanying table:

---

1. Phenolsulphonephthalein.

CLASSIFICATION OF CASES WITH FAILING HEART

| | Male | Female | Total |
|---|---|---|---|
| 1. Rheumatic | 108 | 170 | 278 |
| 2. Syphilitic | 52 | 22 | 74 |
| 3. Arteriosclerotic | 53 | 40 | 93 |
| 4. Nephritic | 59 | 58 | 117 |
| | 272 | 290 | 562 (93% of all) |
| Goiter Heart | 3 | 5 | 8 |
| Doubtful Cases | 19 | 11 | 30 |
| | 294 | 306 | 38 (7% of all) |
| Total | | | 600 |

THE RHEUMATIC TYPE.

Under "rheumatic" are grouped the cases of weakened heart, adherent pericardium and valvular disease associated with (a) acute non-gonorrheal polyarthritis ("rheumatic fever"); (b) chorea (of Sydenham's type); (c) acute tonsilitis, and (d) "primary endocarditis" with negative Wassermann reaction, especially when the cardiac symptoms followed these diseases during the patient's youth. In my opinion it has been demonstrated by the converging results of English, German and American work as the best working hypothesis that a streptococcus is the exciting agent of acute "rheumatism," chorea, endocarditis and tonsilitis.[2] This streptococcus in the joints produces acute rheumatism, in the brain chorea, in the heart endocarditis, myocarditis and pericarditis. It may attack the heart first and later the joints. Then our old terminology would make it appear that endocarditis is the "cause" of rheumatism. It may attack first the brain and heart, appearing later in the tonsillar or peritonsillar tissue. It amy attack the heart and kidney simultaneously, so that we find true cardiac and renal disease from an identical organism, as Libman and others have maintained.

2. As well as certain cases of pleuritis, pericarditis, peritonitis, iritis, meningitis, pneumonia and nephritis.

This form of streptococcosis is essentially a disease of youth, both in man and in the animal experiments of Rosenow. In 60 per cent of the cases here analyzed the disease began (usually in the joints) before the twenty-second year. The patient rarely dies a mechanical or heart-death, usually an infectious death, by terminal, progressive or relapsing infection. Women are affected more often than men—nearly twice as often in these statistics.

The mitral valve, the pericardium and the myocardium are the favorite seats of bacterial invasion, the other valves much less often. In the valves this form of streptococcosis produces usually stenosis with more or less leakage—but almost never leakage alone. In other words, mitral stenosis is the rheumatic (or streptococcic) valve lesion, although it is combined with aortic stenosis in a relatively small percentage of cases. It rarely produces aortic stenosis without a severer stenotic lesion of the mitral. It seldom if ever produces severe symptoms or death by leakage (mitral, aortic or both) alone, though it often produces systolic apical murmurs alone.

On the other hand, it seems probable that stenosis at the mitral or aortic valves involves regurgitation as well in practically every case.

When a cardiac streptococcosis occurs in its most virulent and intractable forms, we call it "malignant," "ulcerative," "septic," or "slow" (endocarditis lenta) and we find in the blood (according to Rosenow) a streptococcus culturally distinguishable from the organism of the milder cases. Nevertheless, I have here included all such cases in the "rheumatic" group of my table.

If the patient survives the second decade of life his smouldering, often recrudescent, cardiac infection may die out, leaving a serviceable heart, despite some narrowing at mitral or even at the mitral and aortic valves. Such cases carry a good prognosis and are often compatible with twenty, thirty or forty years of hard work.

### SYPHILITIC HEART-DISEASE.

Most cases of "causeless" aortic regurgitation appearing in men between 20 and 40 without "rheumatic" history have a positive Wassermann reaction and may be considered due to syphilitic aortitis with or without aneurysm. In negroes and south Italians we find a great deal of this type of heart-disease because of the prevalence of syphilis among them.

I have included twenty-four such cases, together with the eighteen aneurysms, among the syphilitic group in my table. To these I have added a group of thirty-two failing hearts, with or without evidence of valve lesions, without rheumatic history, without evidence of nephritis or arteriosclerosis, but with a positive Wassermann reaction. The permeation of the congenital syphilitic's myocardium with spirochetes (as shown by Warthin and others) makes it probable that in adults as well, many cases of myocarditis are due to syphilis. Probably a more searching study would result in transferring some cases from the arteriosclerotic to the syphilitic column in my table.

### ARTERIOSCLEROTIC HEART-DISEASE.

Under "arteriosclerotic" are included the cases of weakened heart in elderly persons with sclerotic peripheral arteries and hypertension, often with angina pectoris, occasionally with cerebral or peripheral symptoms of the arteriosclerotic type, and always with negative Wassermann reactions. I do not attempt to decide the question whether any of the hearts of this group are weakened solely as a result of renal arteriosclerosis or whether generalized arteriosclerosis works part or all the mischief. In either case arterial disease is the cause. If anemia is present these cases more often belong to the next group.

With further knowledge this arteriosclerotic group could probably be subdivided into the plumbic cases, those of infectious origin, etc. The heart itself shows enlargement, weakness, often arhythmia or gallop-rhythm. Angina pectoris and the cerebral and peripheral evidences of arteriosclerosis are often present. Such patients often live many years in comparative comfort. On the other hand, they may die suddenly of coronary stenosis or cerebral hemorrhage.

NEPHRITIC (OR NEPHROGENIC) HEART-DISEASE.

Volhard and some other modern authors would designate many of my "arteriosclerotic" cases as "benign chronic nephritis." But it seems to me more convenient to confine the term "nephritic or nephrogenic heart-disease" to the cases of weakened and enlarged heart apparently resulting from the hypertension of glomerulonephritis.

Such cases are associated with urinary anamolies more marked than those seen in the arteriosclerotic group. The hyposthenuria, the nocturia, albuminuria, cylindruria and especially the functional weaknesses with the "red test" are very marked. The response to cardiac stimulants and to drugs like theobromin sodium salicylate is feeble. Demonstrable arteriosclerosis of the peripheral vessels is slight or absent. Uremic manifestations, retinitis and anemia are relatively common. The patients of this group average 36 years of age, those of the arteriosclerotic group 59 years of age.

Cases with positive Wassermann reaction or marked history of rheumatism are excluded. The heart itself shows enlargement, an accentuated aortic second sound, sometimes a systolic murmur at the apex or base, often gallop- rhythm and valvular first sound, occasionally an absolute arhythmia. The usual prognosis is for not more than one or two years of life.

Most of the cases of "senile heart," "cardiorenal" disease, "myocarditis' and myocardial weakness have been grouped with the arteriosclerotic cases, some with the nephritic, a few with the syphilitic.

The table shows that 93 per cent of 600 successive and unselected cases of failing heart can be pushed into one of these four groups. How much violence was used in the grouping process I shall try to suggest later on.

